# Ranking and compacting binding segments of protein families using aligned pattern clusters

**DOI:** 10.1186/1477-5956-11-S1-S8

**Published:** 2013-11-07

**Authors:** En-Shiun Annie Lee, Andrew KC Wong

**Affiliations:** 1 Department of Systems Design Engineering, Waterloo, Ontario, Canada

**Keywords:** Protein Analysis, Protein Function Identification, Pattern Discovery, Pattern Clustering, Hierarchical Clustering, Pattern Search, Motif Finding, Local Alignment, Drug Discovery

## Abstract

**Background:**

Discovering sequence patterns with variation can unveil functions of a protein family that are important for drug discovery. Exploring protein families using existing methods such as multiple sequence alignment is computationally expensive, thus pattern search, called motif finding in Bioinformatics, is used. However, at present, combinatorial algorithms result in large sets of solutions, and probabilistic models require a richer representation of the amino acid associations. To overcome these shortcomings, we present a method for ranking and compacting these solutions in a new representation referred to as Aligned Pattern Clusters (APCs). To tackle the problem of a large solution set, our method reveals a reduced set of candidate solutions without losing any information. To address the problem of representation, our method captures the amino acid associations and conservations of the aligned patterns. Our algorithm renders a set of APCs in which a set of patterns is discovered, pruned, aligned, and synthesized from the input sequences of a protein family.

**Results:**

Our algorithm identifies the binding or other functional segments and their embedded residues which are important drug targets from the cytochrome c and the ubiquitin protein families taken from Unitprot. The results are independently confirmed by pFam's multiple sequence alignment. For cytochrome c protein the number of resulting patterns with variations are reduced by 76.62% from the number of original patterns without variations. Furthermore, all of the top four candidate APCs correspond to the binding segments with one of each of their conserved amino acid as the binding residue. The discovered proximal APCs agree with pFam and PROSITE results. Surprisingly, the distal binding site discovered by our algorithm is not discovered by pFam nor PROSITE, but confirmed by the three-dimensional cytochrome c structure. When applied to the ubiquitin protein family, our results agree with pFam and reveals six of the seven Lysine binding residues as conserved aligned columns with entropy redundancy measure of 1.0.

**Conclusion:**

The discovery, ranking, reduction, and representation of a set of patterns is important to avert time-consuming and expensive simulations and experimentations during proteomic study and drug discovery.

## Introduction

**Figure 1:**
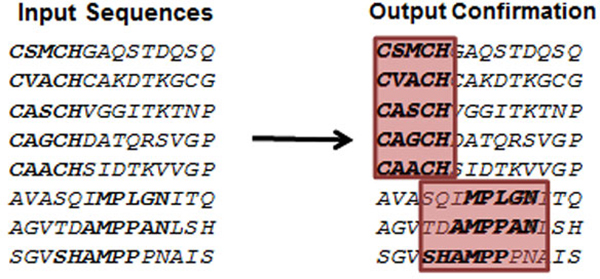
**An intuitive example from the cytochrome c protein showing parts of the protein sequence that represent the binding sites**.

A key concern in healthcare is the major human diseases of the decade, i.e. cancer [1], Alzheimer disease, and SARS [2]. Researchers are critically pursuing solutions to address these diseases. During drug discovery, it is crucial to identify proteins as drug targets and validate their functionality [3]. Binding sites are typically the central function of a protein, and therefore, recognizing them is essential in protein function analysis. Although each protein of the same protein family performs the same function, there are variations amongst the amino acids on each primary sequence. Hence, the conserved amino acid associations on the protein sequences from one protein family reflect important functions. For example, a significant functionality of the cytochrome c protein is to bind the heme ligand from its binding sites [4], and the iron atom in the heme ligand is bonded by two binding residues, one for each side. In our experiments, it was found that each of these two binding residues is contained in a binding segment, which is represented by a sequence pattern with variations. The binding of cytochrome c and its release from the mitochondria has been shown to prevent cell death for cancer treatment [5]. Similarly, the ubiquitin protein contains seven binding residues that are also surrounded by binding segments. These binding residues and segments function by linking individual ubiquitins to create a unique poly-ubiquitin that can be recognized by other ubiquitins. Linking of these binding proteins is directly involved in the control of cancer progression [6]. A common approach to studying a protein family's function is finding the sequence patterns that have variations. Functional patterns can mutate through evolution [7,8]; thus each occurrence of the pattern may not be an exact repeat at the same location. Hence it is difficult to find and locate the segments that embed the binding residues. Figure 1 illustrates simpler patterns that might occur in the consensus region of a protein family. The example contains six text patterns embedded five times each in 30 input sequences and will be referred to as the illustrative example throughout the paper.

Figure 1: An intuitive example from the cytochrome c protein showing parts of the protein sequence that represent the binding sites.

In Bioinformatics, two common approaches for identifying the protein family's function are by multiple sequence alignment and by motif finding. Multiple sequence alignment aligns a set of protein sequences from the same protein family in order to identify important regions and sites in the resulting alignment. Common multiple sequence alignments include Clustal Omega [9], T-Coffee [10], DIALIGN [11] and HMMER [12]. However, finding the global optimal alignment is computationally expensive, and is known in computational complexity as an NP-complete problem [13]. Even with approximate heuristics added, multiple sequence alignment is not efficient in handling large datasets. Moreover, this approach is only appropriate for highly similar sequences, but not for sequences with considerable dissimilarity. Therefore, instead of aligning the entire sequence globally, it is only suitable to identify similarities locally. Thus, the suspected consensus regions have to be located and preprocessed ahead of alignment.

Another approach for identifying the protein family's function by similar local subsequences [14] is called motif finding, which builds motifs into combinatorial models and probabilistic models. The combinatorial model identifies commonly repeated sequence patterns exhaustively [15-17]. Work reported in Pevzner et al. [18] and Mandoiu et al. [19] created cliques where vertices are sequence patterns, edges connect similar sequence patterns, and complete graphs represent the best consensus patterns. However, these combinatorial methods are computationally intensive [20,21] and produce too many possible candidates. The probabilistic model commonly uses the position weight matrix (PWM), which estimates an amino acid at each position while assuming that each position is independent [22,23]. An alternative random sequence synthesis takes further frame-shifted position into consideration by optimally aligning amino acids to create a probabilistic sequence [24,25]. Other probabilistic methods make use of the Markov model, where the current state depends on a specified set of the past states. One such example is the popular pFam database [26], which builds a profile Hidden Markov Model (HMM) from the multiple sequence alignment of a protein family for classifying proteins and predicting their functionality. In general, the probabilistic models compress the data into probability distributions and express amino acid associations as an ordered set of random variables.

To overcome these limitations, we approached the problem from a data mining perspective where we first considered the occurrences and strength of the sequence patterns. We began by identifying a set of statistically strong sequence patterns and developed an Aligned Pattern (AP) Synthesis Process to align and cluster similar patterns into a reduced set of Aligned Pattern Clusters (APCs) for representing the similar sequence patterns that might be associated with binding segments. These APCs capture both the statistically significant sequence association of amino acids as well as their conservations on each of the aligned columns. More precisely, our APC Process aligns and groups similar sequence patterns with variations to form a cluster of Aligned Patterns called APCs. We then examined whether or not the APCs correspond to the binding segment and binding residues that reflect the protein's functionality. This paper is an expansion of *Lee et. al *[27] with an expansion upon reduction and ranking of the results. The three ranking presented are coverage, quality, and standard residual.

When our APC Process was applied to the cytochrome c and ubiquitin protein families, we discovered a reduced set of APCs solutions, which corresponds to the functional binding segments and binding residues of both families. Our APC Process obtained a set of solutions smaller when compared to the combinatorial methods, rendering a more compact yet knowledge-rich representation in the form of the APC than the probabilistic method. Having a smaller set of richer representation is crucial in identifying the drug targets for drug discovery.

## Methodology

**Figure 2:**
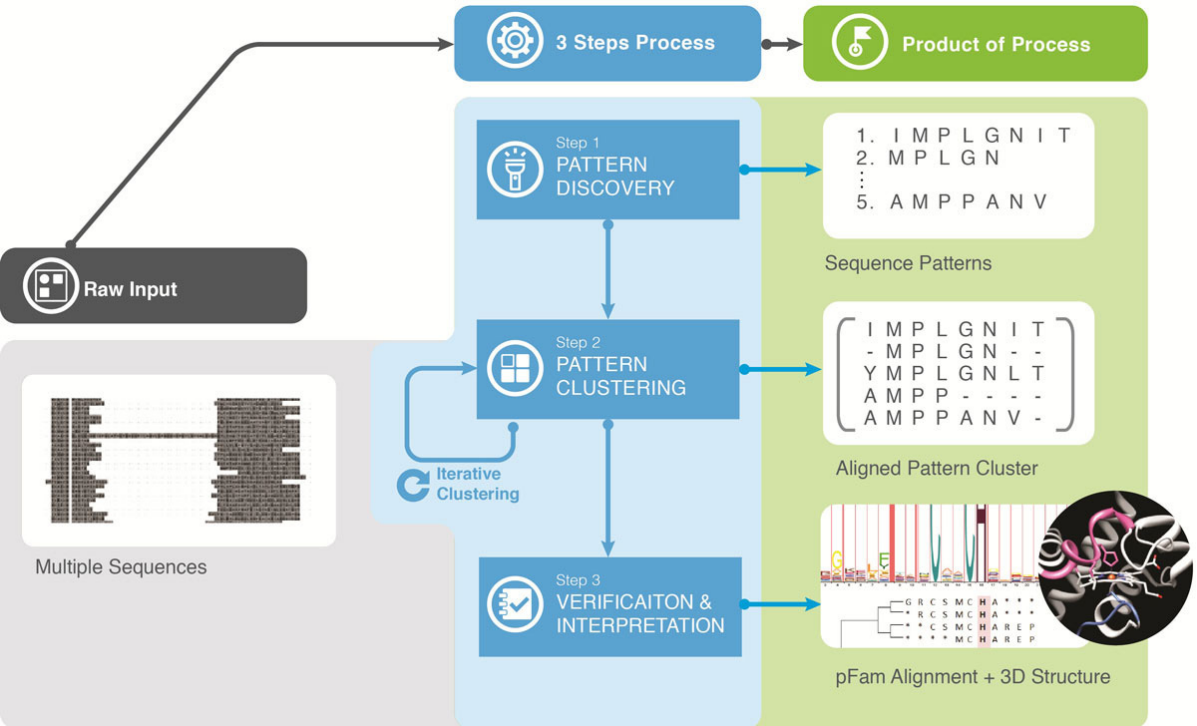
**A text example using the English alphabet illustrates the problem of sequence patterns with variations**. It was created to demonstrate each step of the process succinctly. This text example will be repeated throughout the paper. The overall APC Process contains two steps: the PD Step, and the APC Step. The final result is a list of APCsordered by their ranking.

This section introduces the mathematical notations and definitions required to describe the APC Process and the APC as well as the dual composition of amino acids in the original input data space and in the compact pattern space, both of which are used for calculating measures for revealing pattern characteristics. Our Aligned Pattern Clustering Process, as illustrated by the text example, undergoes two steps (Figure 2): (1) the Pattern Discovery Step (PD Step), and (2) the Aligned Pattern Clustering Step (APC Step). The PD Step discovers the most significant and non-redundant amino acid associations as sequence patterns amongst the family of sequences. Next, the APC Step groups and aligns these discovered patterns into APCs, even though the occurrences of the pattern start at different positions in their input sequences, thus consensus regions do not need to be constrained nor specified. A glossary of terms and mathematical notation is available as Additional file 1 to complement the definitions in the Methodology section.

Figure 2: A text example using the English alphabet illustrates the problem of sequence patterns with variations. It was created to demonstrate each step of the process succinctly. This text example will be repeated throughout the paper. The overall APC Process contains two steps: the PD Step, and the APC Step. The final result is a list of APCsordered by their ranking.

### The input sequences

To begin, the input sequence is built from the alphabet Σ contains a set of characters $\left\{\sigma_{1},\sigma_{2}\ldots ,\sigma_{|\text{Σ}|-1},\sigma_{\left|\text{Σ}\right|}\right\}$. As an example, the English alphabet contains 26 characters, {'a', 'b', ..., 'y', 'z'} = Σ, mathematically, *σ*_1 _='a', *σ*_2 _='b', . . ., *σ*_25 _='y', *σ*_26 _='z', and *|*Σ*| *= 26.

**A single sequence **Let *sk *be a sequence indexed by *k *composed of consecutive elements taken from the alphabet Σ. $s^{k}=s_{1}^{k}s_{2}^{k}\ldots s_{|s^{k}|-1}^{k}s_{\left|s^{k}\right|}^{k}$, where each $s_{i}^{k}\in \text{Σ}$ and *s^k ^*is of length *|s^k^|*. For example, aaaaaaaaaaaaHELLOaaaaaaaaaaaa is a sequence of length 29. This sequence can be represented by *s*1, where *|s*^1^*| *= 29, and the character at position 13 is $s_{13}^{1}=\text{H}$.

**A set of sequences **Let $S=\left\{s^{k}|k=1,\ldots ,|S|\right\}=\left\{s^{1},s^{2},\ldots s^{|S|-1},s^{\left|S\right|}\right\}$ be the set of sequences that represents the set of the input sequences, also called the data space, where |S| is the total number of input sequences, and each sequence having the length of $\left|s^{1}|,|s^{2}|,\ldots ,|s^{|S|-1}|,\;|s^{\left|S\right|}\right|$ respectively. Let each sequence, say sequence *k*, be $s^{k}=s_{1}^{k}\ldots s_{j}^{k}\ldots s_{\left|s^{k}\right|}^{k}$, where *sk *∈ Σ is the elements at position *j *of sequence *k*. Together the data space is the set of sequences is

(1) $$s^{1}=s_{1}^{1}s_{2}^{1}s_{3}^{1}\ldots s_{\left|s^{1}\right|}^{1},$$

(2) $$s^{2}=s_{1}^{2}s_{2}^{2}s_{3}^{2}\ldots s_{\left|s^{2}\right|}^{2},$$

⋮

(4) $$s^{k}=s_{1}^{k}\ldots s_{j}^{k}\ldots s_{\left|s^{k}\right|}^{k},$$

⋮

(6) $$s^{\left|S\right|}=s_{1}^{\left|S\right|}s_{2}^{\left|S\right|}s_{3}^{\left|S\right|}\ldots s_{\left|s^{\left|S\right|}\right|}^{\left|S\right|},$$

### The pattern discovery step

The PD Step is a previously developed pattern discovery and pruning algorithm [28] that obtains a condennse list of significant patterns from the family of protein sequences.

**The pattern **In this paper, we consider a pattern as a statistically significant and non-redundant pattern as defined in Wong et. al [28].

**Definition 1 ***A pattern ${\bar{p}}^{i}=s_{ji}^{i}s_{ji+1}^{i}\ldots s_{ji+|{\bar{p}}^{i}|-1}^{i}$ is a short sequence over *Σ *where $\left|{\bar{p}}^{i}\right|$ is its order (or length). The sequence association is statistically significant and non-redundant in the sense that it is deltaclosed (i.e. it is not covered by a statistically significant super-pattern) and non-induced, (i.e. its statistical significance is not induced by its statistically strong sub-patterns ). A pattern *${\bar{p}}^{i}$*is discovered by passing four statistical conditions defined in Wong et. al *[28].

*An *UNALIGNED PATTERN ${\bar{p}}^{i}$*is discovered by passes four statistical conditions defined in Wong et. al *[28].

An occurrence of the pattern ${\bar{p}}^{i}$ is expressed as $occ\left({\bar{p}}^{i}\right)=j_{i}$ such that ${\bar{p}}^{i}=s_{j_{i}}^{i}s_{j_{i}+1}^{i}\ldots s_{j_{i}+|{\bar{p}}^{i}|-1}^{i}$,

Where *i *is the index of the sequence in which that pattern occurs, and *j_i _*is the starting index in that sequence *si *where the pattern begins.

(7) $$s^{1}=s_{1}^{1}\ldots s_{j_{1}+1}^{1}s_{j_{1}+2}^{1}\ldots s_{j_{1}+|{\bar{p}}^{i}|-1}^{1}s_{j_{1}+|{\bar{p}}^{i}|}^{1}\ldots s_{\left|s^{1}\right|}^{1}$$

(8) $$s^{2}=s_{1}^{2}\ldots s_{j_{2}+1}^{2}s_{j_{2}+2}^{2}\ldots s_{j_{2}+|{\bar{p}}^{i}|-1}^{2}s_{j_{2}+|{\bar{p}}^{i}|}^{2}\ldots s_{\left|s^{2}\right|}^{2}$$

…

(10) $$s^{\left|S\right|}=s_{1}^{\left|S\right|}\ldots s_{j_{\left|S\right|}+1}^{\left|S\right|}s_{j_{\left|S\right|}+2}^{\left|S\right|}\ldots s_{j_{\left|S\right|}+|{\bar{p}}^{i}|-1}^{\left|S\right|}s_{j_{\left|S\right|}+|{\bar{p}}^{i}|}^{\left|S\right|}\ldots s_{m}^{\left|S\right|}$$

The text example (Table 1) displays two patterns corresponding to our definition. The dataset contains two functional patterns, HELLO and MELLOW, are English words embedded in ten input sequences $S=\left\{s^{1},\ldots ,s^{10}\right\}$. The letters outside the patterns are stochastically generated from the 26 characters in the English alphabet that are identically and independently distributed.

**Table 1:** Example of patterns ${\bar{p}}^{1}$ =HELLO and ${\bar{p}}^{2}$ =MELLOW.

S	The Input Sequences
*s*^1^	bdxejrtewkwkHELLOkcmstsjavtpi
*s*^2^	nfixtHELLOuzdovcaaxnkjfjcvwk
*s*^3^	dimtndvkjmkHELLObkcmstsj
*s*^4^	tzhgarzofdHELLOpwkxmc
*s*^5^	tyjxjqnyHELLOwmopemlqfgptnwnq
*s*^6^	kntywtoaxMELLOWbtiasycma
*s*^7^	jilxchitivMELLOWriiiweyfzgvuyaa
*s*^8^	hmlzvMELLOWorgfeb
*s*^9^	xhmlzvqgcanyMELLOWgbfj
*s*^10^	vqgcanyffcMELLOWvcnsnjvalbdvr

**Data induced by the unaligned pattern** Let $D\left({\bar{p}}^{i}\right)$, be each of the unique occurrences of the pattern, ${\bar{p}}^{i}$, found in the input sequence. We call $D\left({\bar{p}}^{i}\right)$ the data induced by ${\bar{p}}^{i}$ or the induced data of ${\bar{p}}^{i}$. We will return to the concept on the data induced by pattern when we use it to compute the measures for aligned columns within the context of APC.

### The aligned pattern clustering step

For the APC Step, we developed an algorithm that gathers a set of similar patterns of different lengths obtained from the PD Step while aligning them into patterns of the same length by inserting gaps and wildcards. Constrained by the statistical sequence association, the corresponding elementNames in this cluster of patterns are lined up into columns, thus reflecting their conservation and variation [27].

**Figure 3:**
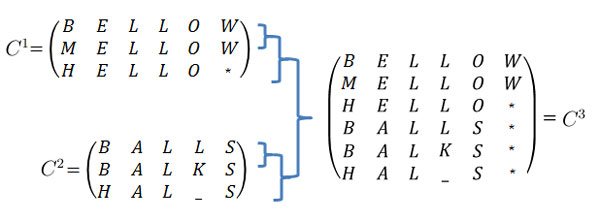
**In one iterative step of hierarchical clustering, an existing APC, *C*_1 _with *m *= 3 and *n *= 6, is merged with another APC, *C*_2 _with *m *= 3 and *n *= 5, to result in the new APC, *C*_3_, which is extended to *m *= 6 and *n *= 6**. (a) Binding Segments (b) Binding Residues

In this paper, the APC Step is a single-linkage hierarchical clustering algorithm that takes an input of a list of patterns and synthesizes, or more precisely aligns and groups, the patterns into one or more APC(s) based on a similarity measure between APCs. Using the text example, Figure 3 illustrates one iteration of the hierarchical clustering algorithm. More precisely, it shows the last step of the iterative merge between APC *C*_1 _and APC *C*_2_, thereby creating the new APC *C*_3_.

Figure 3: In one iterative step of hierarchical clustering, an existing APC, *C*_1 _with *m *= 3 and *n *= 6, is merged with another APC, *C*_2 _with *m *= 3 and *n *= 5, to result in the new APC, *C*_3_, which is extended to *m *= 6 and *n *= 6.

**Definition 2 ***A set of *$APC\;\mathbb{C}=\left\{C^{l}|l=1,\ldots ,|\mathbb{C}|\right\}=\left\{C^{1},C^{2},\ldots ,C^{|\mathbb{C}|-1},C^{\left|\mathbb{C}\right|}\right\}$

An APC, C^l^, is a set of similar horizontal sequence patterns that have been optimally grouped and vertically aligned into a set of patterns ${\mathbb{P}}^{l}=\left\{p^{1},p^{2},\ldots p^{m}\right\}$ represented by C^l^, which is expressed as

(12) $$C^{l}=ALIGN\left({\mathbb{P}}^{l}\right),$$

(13) $$={\left(\begin{array}{cccc} s_{1}^{1} & s_{2}^{1} & \ldots & s_{n}^{1}\\ s_{1}^{2} & s_{2}^{2} & \ldots & s_{n}^{2}\\ \vdots & \vdots & \vdots & \vdots \\ s_{1}^{m} & s_{2}^{m} & \ldots & s_{n}^{m} \end{array}\right)}_{m\times n}=\left(\begin{array}{c} p^{1}\\ p^{2}\\ \vdots \\ p^{m} \end{array}\right),$$

(14) $$=\left(\begin{array}{cccc} c_{1} & c_{2} & \ldots & c_{n} \end{array}\right).$$

where $s_{j}^{i}\in \text{Σ}\cup \left\{-\right\}\cup \left\{*\right\}$ is an amino acid in the pattern, p^i^, in an aligned column j. Each patterns of C^l ^is aligned into length |C^l^| = n, and there is a set of $|{\mathbb{P}}^{l}|\;=m$ patterns (rows) in C^l^.

In the text example in Figure 3, *C*_1 _with *m *= 3 and *n *= 6, is merged with another APC, *C*_2 _with *m *= 3 and *n *= 5, to result in the new APC, *C*_3_, which is extended to *m *= 6 and *n *= 6.

**Definition 3 ***An Aligned Pattern, which will simply be referred to as a pattern from this point forward, is a sequence of order-preserving amino acids maximizing the similarity of the patterns against a set of pattern from an APC, ${\mathbb{P}}^{l}$ of size *$|{\mathbb{P}}^{l}|\;=m$*with gaps, wildcards, and mismatches. Let $p^{i}\in {\mathbb{P}}^{l}$ be $s_{1}^{i}s_{2}^{i}\ldots s_{\left|p^{i}\right|}^{i}$, where *$s_{j}^{i}\in \text{Σ}\cup \left\{-\right\}\cup \left\{*\right\}$*is an amino acid in the pattern pi and in the aligned column index c_j_*.

**Definition 4 ***An *aligned column *cj in C^l ^represents the j^th ^column of amino acids that have been aligned from the set of patterns contained in the current APC, C^l ^*= ( *c*_1 _*c*_2 _*… c_n_*)*. A conserved *ALIGNED COLUMN*is conserved to only one type of amino acid such that c_j _*= [*σ ... σ ... σ*]*^T ^where σ *∈ Σ.

For the text example, the APC Step creates an APC containing six patterns with six aligned columns (Table 2). The APC is obtained from the alignment of a set of similar patterns, where each row is a pattern from ${\mathbb{P}}^{l}$ and each column is an aligned column of amino acids. Here, the pattern for the third row is *p*^3 ^= *HELLO *and the aligned column for the first position is *c*_1 _= [*BM HBBH*]*^T^*

**Table 2:** Example of an APC for the text example.

*p^i^*\*c_j_*		${\left(c1\;c2\;c3\;c4\;c5\;c6\right)}_{1\times 6}$
${\left(\begin{array}{c} p1\\ p^{2}\\ p^{3}\\ p^{4}\\ p^{5}\\ p^{6} \end{array}\right)}_{6\times 1}$	=	${\left(\begin{array}{c} B\;\;E\;L\;L\;O\;W\\ M\;E\;L\;L\;O\;W\\ H\;E\;L\;L\;O\;*\\ B\;A\;L\;L\;\;S\;*\\ B\;A\;L\;K\;\;S\;*\\ H\;A\;L\;-\;\;S\;* \end{array}\right)}_{6\times 6}$

**Data induced by apc **Let $D\left(C^{l}\right)$ be data induced by the APC *C^l^*, which is the subset of segments from the input sequences, or the data subspace containing all the pattern from the APC, *Cl*, where its corresponding ${\mathbb{P}}^{l}={\left\{p^{1},p^{2},\ldots p^{m}\right\}}^{T}$. We call $D\left(C^{l}\right)$ the data induced by *C^l ^*or the induced data of *C^l^*. Then $D\left(C^{l}\right)$ is then the union of the segements from the input sequences induced by all the patterns contained in *C^l^*, $D\left(C^{l}\right)=D\left(p^{1}\right)\cup D\left(p^{2}\right)\cup \cdots \cup D\left(p^{m}\right)=\underset{\forall pi\in {\mathbb{P}}^{l}}{⋃}D\left(p^{i}\right)$

### Measuring and ranking results

#### The three measures of APCs

In order to rank the set of constructed APCs,  ℂ, three measures are computed for each APC, *C^l^*. The three measures are Coverage, APC Quality, and Standard Residual.

**Coverage **The coverage of an APC accounts for the total input sequences that are covered by the APC, *C^l^*, over the entire set of input sequences. Note that this is also counting the number of occurrences in the induced dataspace $D\left(C^{l}\right)$.

**APC Quality **The APC Quality, *Q*, is the average column entropy subtracted from one, where entropy is computed from the set of Aligned Patterns, ${\mathbb{P}}^{l}\in C^{l}$. The APC Quality measures the stability or reliability of a APC, whereas the entropy measures the randomness or the degree of variation within an APC. The value of *Q *approaches one while the resulting APC is more stable. The value of *Q *approaches zero while the resulting APC is more random. *Q *is expressed as:

(15) $$Q=1-\frac{\overset{n}{\underset{j=1}{\sum }}H\left(c_{j}\right)}{n},$$

where *cj *is the aligned column in the resulting APC.

(16) $$H\left(c_{j}\right)=-\underset{\forall \sigma \in c_{j}}{\sum }Pr\left(c_{j}=\sigma \right)\text{log}Pr\left(c_{j}=\sigma \right),$$

(17) $$Pr\left(c_{j}=\sigma \right)=\frac{\overset{m}{\underset{i=1}{\sum }}1\left(s_{i}^{j}=\sigma \right)}{m}$$

where *σ *∈ Σ ∪ {−} ∪ {*} is the amino acid $s_{i}^{j}$ of *p_i _*at *c_j_*, and the probability *Pr*(*c_j _= σ*) is computed from counting the subset of patterns in ℙ*^l^*.

**Standard Residual **The Standard Residual measures the statistical significance of the APC by comparing the actual number of occurrences, *o*, of all the patterns included in the APC, against the expected number of occurrences, *e*, which is computed from the default random model of APC. It is written as

(18) $$\text{StandardResidual }=\frac{o-e}{\sqrt{e}},$$

where *o *is the actual number of occurrences of the pattern in P*^l ^*counted from the input data, $D\left(C^{l}\right)$ and *e *is the expected number of occurrences computed from the default random model of APC, *C*, by assuming that each of the aligned columns *c_j _*are independent and identically distributed (i.i.d.) shown below:

(19) e=E[C],

(20) $$=N\left(Pr\left(C\right)\right),$$

(21) $$=N\left(Pr\left(c_{1}\right)Pr\left(c_{2}\right)\ldots Pr\left(c_{n}\right)\right),$$

(22) $$=N\left(\overset{n}{\underset{j=1}{\prod }}Pr\left(c_{j}\right)\right),$$

where *N *is the length of the input sequence and each of the aligned columns *c_j _*∈ *C *is i.i.d. To compute the default probability of the aligned columns, *Pr*(*cj*), sum the probability of all the possible amino acids in the one single aligned columns. $Pr\left(c_{j}\right)=Pr\left(c_{j}=\sigma_{\text{1}}\right)+Pr\left(c_{j}=\sigma_{\text{2}}\right)+\cdots +Pr\left(c_{j}=\sigma_{k}\right)=\underset{\forall \sigma_{k}\in c_{j}}{\sum }Pr\left(c_{j}=\sigma_{k}\right)$, where $Pr\left(c_{j}=\sigma_{k}\right)=\frac{1}{20}$ for each *σ_k _*is i.i.d. Returning to the text example with 6 patterns and 6 aligned columns and the English alphabet, $Pr\left(c_{\text{1}}\right)=Pr\left(c_{\text{1}}=B\right)+Pr\left(c_{\text{1}}=M\right)+Pr\left(c_{\text{1}}=H\right)\;=\frac{3}{26}$. Therefore, the final expectation is

(23) $$e=N\left(\overset{n}{\underset{i=1}{\prod }}\left(\underset{\forall \sigma_{k}\in c_{j}}{\sum }Pr(c_{j}=\sigma_{k}\right)\right).$$

#### The redundancy measure of the aligned columns

The Redundancy Measure [29] indicates the specificity or stability of the amino acids in an aligned column based on the frequency of the occurrences of the amino acids taken from that aligned column of its $D\left(C^{l}\right)$. The Redundancy Measure *R*1(*cj *) for the aligned column *cj *is

(24) $$R\text{1}\left(c_{j}\right)=\text{1}-H\left(c_{j}\right).$$

where *H*(*c_j_*) with *Pr*(*c_j _= σ*) being computed from counting of *σ *in the aligned column, *c_j_*, of the entire input sequences, $D\left(C^{l}\right)$. Hence, a conserved aligned column has *R*1(*c_j_*) = 1 since minimum entropy value of *H*(*c_j_*) = 0. Similarly a variable aligned column has *R*1(*c_j_*) = 0 the maximum entropy value of *H*(*c_j_*) = 1. If the amino acid occurrences in $D\left(C^{l}\right)$ are equiprobable.

Note that the entropy of the Redundancy Measure is computed from the entropy of the induced data, $D\left(C^{l}\right)$, whereas the entropy of the APC Quality uses the amino acids from the patterns in ${\mathbb{P}}^{l}$. This is because the quality of the APC measures how much variation or stability is in the patterns, whereas the redundancy of the aligned column measures how much the redundancy or consistency is in the induced data.

## Results and discussions

We applied our APC Process on the cytochrome c and the ubiquitin protein families in order to examine how the resulting APCs relate to the binding sites, which are the biologically significant regions of the protein. There are three aspects we would like to explore: the reduction of the set of candidate solutions from the discovered patterns to APCs obtained; how each pattern in the APC surrounding the binding site represents a binding segment in a single strand of protein; and how binding residues correlate to their aligned column. Finally, we display our results underneath the pFam multiple sequence alignment to compare the differences in the representations. In the comparison, we demonstrate the overall hierarchical clustering performance of our APC Process as well as the quality of the resulting APCs.

### Cytochrome C results

First, we demonstrated that by grouping similar patterns together, the APC reduces the number of candidate solutions to be examined without losing information. Next, we showed that in the binding APCs, each pattern represents a binding segment in the protein sequence and each of the two binding sites is represented by a specific aligned column. The 317 sequences from the cytochrome c protein family were obtained on September 17th, 2012 from Uniprot by searching the following terms: cytochrome c; AND reviewed:yes; AND name:c*; AND mnemonic:c*; AND (name:cytochrome AND name:c); NOT name:type; NOT name:VPR; NOT name:biogenesis; NOT name:*ase; NOT (name:cytochrome AND name:b*); NOT like; NOT proba*; AND fragment:no; AND active:yes. These selected parameters should help to yield a reasonable number of input sequences for the APC Process. From these 317 input sequences, the PD Step was executed with the *minimal order *of 5, the *minimum occurrence *of 20, and the *delta *of 0.9. The PD Step discovered 154 patterns from the cytochrome c protein family, where 28 patterns, or 18.18% of the total patterns, contain the proximal binding site, His18, and 23 patterns, or 14.94% of the total patterns, contain the distal binding site, Met62, resulting in a combined total of 33.12% of the discovered patterns that contains one of the two binding sites. Therefore, the set of patterns redundantly covers the two binding sites. This observation indicates that each individual pattern alone covers only a small fraction of the input sequences in the data space; therefore, a single pattern by itself cannot fully represent the rich variations of all the input sequences within the entire protein family. Hence, the APC, which contains a set of similar patterns that has been grouped and aligned to allow variations, provides a reduced and much richer representation of the binding segments and binding residues.

In the APC Step, we showed that our APC Process reduced the number of candidate solutions without losing any information and richly captured the binding sites in the compact APCs where the binding segments are the patterns therein and the binding sites are the conserved aligned columns. We ensure that all the patterns discovered are strongly statistically significant by starting with a tighter configuration to ensure the quality of the result. From this list of 154 statistically significant and non-redundant patterns obtained from the previous PD Step, the APC Step was executed with the following settings: the Merge *Algorithm *as Global Alignment, the SIMILARITY*Score *as Hamming Distance, the TERMINATION*Condition *Score less than 0.8, the heuristics column distribution score greater than 0.8 and the minimum of three overlapping column matches.

We found the following two results (Table 4): five APCs (13.89% of the total number of APCs) discovered contains the proximal binding site, His18; five APCs (13.89% of the total number of APCs) contains the distal binding site, Met62; and 27.78% of the combined total. This observation indicates that, while retaining the full information, the 154 patterns were reduced to 36 APCs, a total reduction of 76.62% for documentation and visualization.

**Table 3:** The 36 APCs of the Cytochrome C Family Ranked by Standard Residual (where *m *= the number of patterns in the APC, and *n *= length of the APC)).

	APC (as regular expressions)	*m*	*n*	Quality	Coverage	Standard Residual	Binding Site
1	WGEDTLMEYLENPKKYIPGTK**M**IFAGIKKK	8	30	0.57	81	5.92E+16	Met62
2	MGDVEKGKKIFVQ[KR]CAQC**H**TVEKGGKHKTGPNL	19	33	0.43	119	5.04E+16	His18
3	QC**H**TVEKGGKHKTGPNLHGLFGRKTGQA	7	28	0.41	46	8.32E+14	His18
4	TLYDYLLNPKKYIPGTK**M**[VA]FPGLKKPQ	8	27	0.44	116	1.91E+14	Met62
5	GAGHK[QVT]GPNL[NH]GLFGRQSGTT	13	21	0.4	125	3.53E+10	
6	GFSYTDANKNKGITWGE	8	17	0.41	66	6.33E+08	
7	GEKIFKTKCAQC**H**TV	3	15	0.57	24	6.45E+07	His18
8	MGDVEKGKKIFVQKC	7	15	0.4	53	5.04E+07	
9	GPNLHGLFGRKTGQA	4	15	0.43	46	4.37E+07	
10	ERADLIAYLK[KE]ATNE	9	15	0.4	91	3.53E+07	
11	HGLFGRKTGQAPGF	9	14	0.46	70	2.10E+07	
12	IPGTK**M**AFGGLKK	4	13	0.42	136	9.06E+06	Met62
13	AANKNKGITWGE	4	12	0.5	54	1.60E+06	
14	LHGLFGR[QK]SGTT	6	12	0.42	88	1.07E+06	
15	AGYSYSAANKN	5	11	0.43	30	1.40E+05	
16	TLYDYLLNP	2	9	0.56	29	2.69E+04	
17	GQAPGFSY	2	8	0.5	27	5.57E+03	
18	TK**M**VFAG	2	7	0.57	52	3.38E+03	Met62
19	GGKHKTG	2	7	0.43	64	2.94E+03	
20	EKGKKIF	2	7	0.43	62	2.85E+03	
21	FAGLKKP	3	7	0.48	57	2.62E+03	
22	WGGGKIY	2	7	0.71	27	2.48E+03	
23	FAGIKKK	2	7	0.43	51	2.34E+03	
24	YLKKAT	1	6	1	29	1.19E+03	
25	WGEDTL	1	6	1	25	1.02E+03	
26	NCAAC**H**	2	6	0.83	30	8.68E+02	His18
27	KGAGHK	2	6	0.83	26	7.52E+02	
28	KGITW	1	5	1	49	4.46E+02	
29	GFSYT	1	5	1	42	3.83E+02	
30	FVQKC	1	5	1	39	3.55E+02	
31	DANKN	1	5	1	34	3.10E+02	
32	GYSYT	1	5	1	28	2.55E+02	
33	A**M**PAF	1	5	1	24	2.19E+02	Met62
34	C**H**AGG	1	5	1	22	2.00E+02	His18
35	FKTRC	1	5	1	20	1.82E+02	
36	LFEYL	1	5	1	20	1.82E+02	

**Table 4:** Comparing the Number of APCs and Patternss.

	Patterns Count	%overall	APCs Count	%overall	%Reduction
His18	28	18.18%	5	13.89%	82.14%
Met62	23	14.94%	5	13.89%	78.26%

Total	154	33.12%	36	27.78%	76.62%

It can be seen in Table 5, the top four resulting APCs correspond to the proximal and distal binding segments of the cytochrome c protein family. More specifically, 26 proximal patterns were reduced to the two top APCs (a 92.31% reduction) and 16 distal patterns are reduced to the two top APCs (a 87.50% reduction), for a combined reduction of 88.10% for these top four APCs.

**Table 5:** Comparing the Top Four APCs and their Patterns.

	Pattern Count	APCs Count	%Reduction
His18	26	2	92.31%
Met62	16	2	87.50%

Total	42	4	88.10%

### Cytochrome C discussion

**Figure 4:**
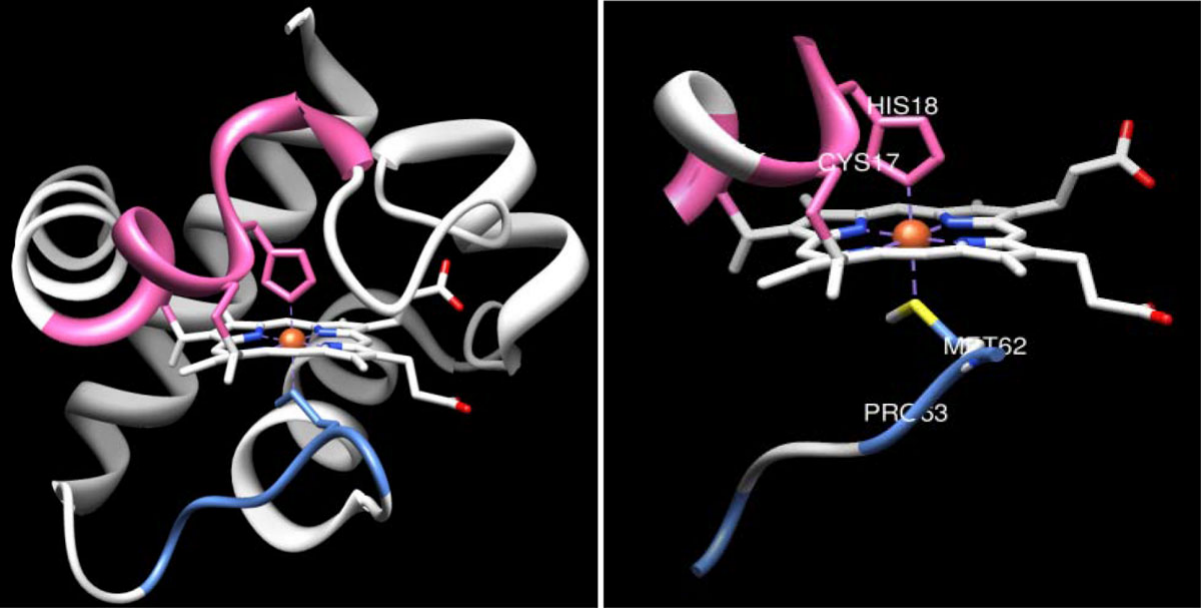
**One three-dimensional structure from the cytochrome c protein family, PDB ID 1F1F, is displayed**. The top-two statistically significant APCs from the cytochrome c protein are the proximal binding segment (in pink) and the distal binding segment (in blue) that bind the heme from above and below the horizontal plane, respectively. More specifically, one specific amino acid from each of the two segments binds the iron molecule from the centre of the heme: the "H" (Histidine) residue at position 18 of the proximal segment and the "M" (Methionine) residue at position 62 of the distal segment.

Biologically, the two binding residues in the cytochrome c protein that bind the heme ligand are the proximal binding residue that binds the heme ligand from the proximal side of the protein [4,30]; and the distal binding residue that binds the heme from the other side of the protein [31]. The proximal and distal binding sites are located in the protein and bind the heme ligand from above and below the horizontal plane, respectively. Specifically, one particular amino acid from each of the two protein segments binds the iron molecule located in the centre of the heme: the "H" (Histidine) residue at position 18, which is the proximal side of the protein sequence and the "M" (Methionine) residue at position 62, which is the distal side of the protein sequence. These two binding residues, His18 and Met62, are also confirmed by the three-dimensional structure, PDBID 1F1F, of the cytochrome c protein (Figure 4). Our results showed that the set of APCs discovered by our APC Step that contained the protein binding sites - the main biological function of the protein. In fact, the four top resulting APCs precisely correspond to these crucial binding segments that contain conserved aligned columns corresponding to the binding residues.

Figure 4: One three-dimensional structure from the cytochrome c protein family, PDB ID 1F1F, is displayed. The top-two statistically significant APCs from the cytochrome c protein are the proximal binding segment (in pink) and the distal binding segment (in blue) that bind the heme from above and below the horizontal plane, respectively. More specifically, one specific amino acid from each of the two segments binds the iron molecule from the centre of the heme: the "H" (Histidine) residue at position 18 of the proximal segment and the "M" (Methionine) residue at position 62 of the distal segment.

The ten APCs that correlate to the two binding sites were first clustered based on their horizontal patterns in their rows and are then aligned into their aligned columns that reveal their vertical stability. Firstly, each APC contains a set of conserved patterns that are similar to one another. Although these patterns suggest their horizontal significance in the protein family, individually they do not identify the significance of the amino acid's conservation and variation. Thus, the aligned columns is important for identifying the stability of the binding residue. Secondly, the aligned columns of each binding APC show the conservation of aligned columns in the cluster, which otherwise is not easily seen in the individual non-variable patterns. For example, consider the top two APCs that correspond to each of the proximal (Table 7) and distal binding segments (Table 6). In the Tables, the columns in bold are the conserved aligned columns with R1 = 1.0, where R1 reflects the specificity of the residue of the site in the APC. The aligned columns corresponding to the binding sites of the APCs have an R1 value of 1.0, that is, the amino acid for that aligned column is conserved in the data space. To give a precise example, consider the proximal APC that is ranked second. This APC has three conserved aligned columns with R1 value of 1.0: Gln16, Cys17, and His18. The His18 conserved aligned column is the proximal binding residue, and the Cys17 binds an adjacent corner on the heme ligand. Similarly, the conserved aligned column representing Met62 in the distal APCacts as the distal binding residue. The other conserved aligned columns can be used to identify other important functions in the protein.

**Table 6:** The Distal APC of the Cytochrome C Family.

**patterns**	**Count**	**Score**
WGEDTLMEYLENPKKYIPGTKMIF******	22	1.94E+03
***DTLMEYLENPKKYIPGTKM********	26	1.30E+03
*******EYLENPKKYIPGTKMIFAGIKK*	35	2.54E+02
****TLMEYLENPKKYIPGTKMIFAGIKKK	29	7.34E+02
****TLMEYLENPKKYIPGTKMIFAG****	34	4.81E+01
********YLENPKKYIPGTKM********	81	6.51E+02
*******EYLENPKKYIPGTKMIFAG****	42	5.44E+01
*******EYLENPKKYIPGTKM********	65	2.88E+01

**Table 7:** The Proximal APC of the Cytochrome C Family.

**patterns**	**Count**	**Position**
******GKKIFVQKCAQCHTV*********	23	6.27E+04
****EKGKKIFVQKCAQCHT**********	23	1.32E+04
MGDVEKGKKIFVQKCAQCHTVEKGGKHKTG	20	7.50E+07
******GKKIFVQKCAQCHTVEKGGKHKTG	20	1.16E+06
*************KCAQCH***********	57	1.59E+01
**************CAQCH***********	89	2.58E+03
*************RCAQCHT**********	21	1.38E+01
**************CAQCHT**********	76	3.01E+01
**********FVQKCAQCHTVE********	27	5.88E+02
************QKCAQCHT**********	32	6.38E+01
************QKCAQCHTVEKGGKHKTG	23	6.33E+04
*************KCAQCHTVEKG******	30	4.91E+01
*************KCAQCHTV*********	51	1.73E+01
**************CAQCHTV*********	65	3.10E+01
**************CAQCHTVEK*******	34	1.30E+01
**************CAQCHTVE********	49	2.41E+01
****************QCHTV*********	95	2.33E+03
****************QCHTVEKGG*****	45	1.75E+01
****************QCHTVE********	77	3.15E+01

**Figure 5:**
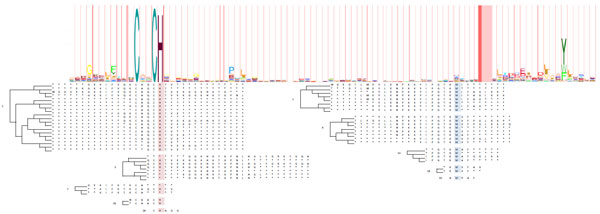
**Ten resulting APCs representing the proximal and distal binding segments of the cytochrome c are compared to the HMM logo from pFam**. In the largest APC, Cys17 is identified as one of the conserved aligned columns, where His18 binds to the heme iron. In the second largest APC, Met62 is identified as one of the conserved aligned column of the distal binding segment, where Met62 binds the heme iron.

By matching the individual APCs up to the independent HMM alignment of pFam (Figure 5), we confirmed the validity of our set of 36 APCs. In addition, our proximal APC for cytochrome c is consistent with the proximal binding motif: [C]-x(2)-[CH], from PROSITE (PDOC00169) [32,33] and a strong emission probability in pFam (PF00034) [26,34]. Moreover, our method strongly identified the distal binding in our APCs where PROSITE does not annotate the binding site and pFam identifies only as a weak emission probability.

In conclusion, the APC can represent protein functions such as the binding segments and binding residues and presents a reduced set of candidate solutions and specifies their location in the protein family. In cytochrome c, the prevention of binding can block cancer progression, which is an important drug discovery for cancer treatment.

### Ubiquitin results

To further study the APC Step, we closely examined the iterative steps and its resulting APCs using the ubiquitin protein family. The 70 sequences from the ubiquitin protein family used in our experiment were obtained on August 9th, 2012 from Uniprot by searching the following terms: name:ubiquitin; NOT name:*ase; NOT name:like; NOT name:ribosomal; NOT name:modifier; NOT name:factor; NOT name:protein; NOT name:conjugating; NOT name:activating; NOT name:enzyme; AND reviewed:yes; AND mnemonic:UB*.

Figure 5: Ten resulting APCs representing the proximal and distal binding segments of the cytochrome c are compared to the HMM logo from pFam. In the largest APC, Cys17 is identified as one of the conserved aligned columns, where His18 binds to the heme iron. In the second largest APC, Met62 is identified as one of the conserved aligned column of the distal binding segment, where Met62 binds the heme iron.

**Figure 6:**
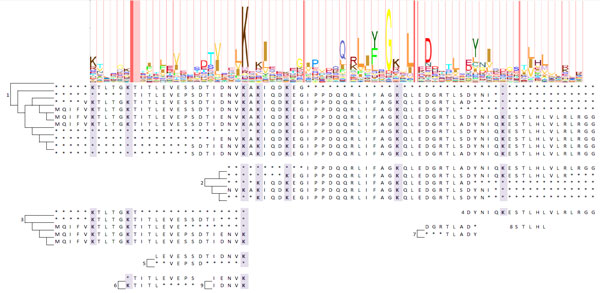
**The seven Lys binding residues of the ubiquitin protein family are highlighted in the APCs: Lys6, Lys11, Lys27, Lys29, Lys33, Lys48, and Lys63**. Six of the seven binding sites are discovered, all except Lys29, are conserved aligned column with R1 = 1.0.

Figure 6: The seven Lys binding residues of the ubiquitin protein family are highlighted in the APCs: Lys6, Lys11, Lys27, Lys29, Lys33, Lys48, and Lys63. Six of the seven binding sites are discovered, all except Lys29, are conserved aligned column with R1 = 1.0.

These adopted parameters help yield a reasonable number of input sequences for our study. From these 70 input sequences, the PD Step was executed with the *minimal order *of 10, the *minimum occurrence *of 20, and the *delta *of 0.9 to yield a proper size of the results for the study. Table 8 shows the thirty discovered patterns, where all except five of the patterns contained the seven binding residues. Nevertheless, these patterns still corresponded to the conserved amino acids around the binding residues. Therefore, all the discovered patterns indicate important functionality in the ubiquitin protein family, such as the binding site or the areas next to the binding site. Once again, each pattern on its own occurs only a few times, and has only a low frequency count for representing the binding segments of this protein family. Since protein binding segments exhibit considerable variability, APCs represent the protein family's functional binding sites more explicitly and effectively.

**Table 8:** Statistically Ranked Patterns Discovered from the Sequences of the ubiquitin Family.

Ranking	Pattern	Frequency	Score	Binding Residue
1	MQIFV**K**TLTG**K**TITLEVEPSDTIENV**K**A**K**I	21	5.44E+44	Lys6, Lys11, Lys27,
	QD**K**EGIPPDQQRLIFAG**K**QLEDGRTLSDYN			Lys29, Lys33, Lys48,
	IQ**K**ESTLHLVLRLRGG			Lys63
2	MQIFV**K**TLTG**K**TITLEVESSDTIDNV**K**A**K**I	15	2.86E+44	Lys6, Lys11, Lys27,
	QD**K**EGIPPDQQRLIFAG**K**QLEDGRTLADYN			Lys29, Lys33, Lys48,
	IQ**K**ESTLHLVLRLRGG			Lys63
3	SDTIENV**K**A**K**IQD**K**EGIPPDQQRLIFAG**K**Q	24	1.25E+33	Lys27, Lys29, Lys33,
	LEDGRTLSDYNIQ**K**ESTLHLVLRLRGG			Lys48, Lys63
4	SDTIDNV**K**A**K**IQD**K**EGIPPDQQRLIFAG**K**Q	17	7.59E+32	Lys27, Lys29, Lys33,
	LEDGRTLADYNIQ**K**ESTLHLVLRLRGG			Lys48, Lys63
5	MQIFV**K**TLTG**K**TITLEVESSDTIDNV**K**A**K**I	17	4.76E+31	Lys6, Lys11, Lys27,
	QD**K**EGIPPDQQRLIFAG**K**QLEDGRTL			Lys29, Lys33, Lys48
6	IENV**K**A**K**IQD**K**EGIPPDQQRLIFAG**K**QL	32	3.48E+31	Lys27, Lys29, Lys33,
	EDGRTLSDYNIQ**K**ESTLHLVLRLRGG			Lys48, Lys63
7	V**K**TLTG**K**TITLEVESSDTIDNV**K**A**K**IQD	17	1.59E+30	Lys6, Lys11, Lys27,
	**K**EGIPPDQQRLIFAG**K**QLEDGRTLAD			Lys29, Lys33, Lys48
8	TITLEVEPSDTIENV**K**A**K**IQD**K**EGIPPD	24	8.80E+28	Lys27, Lys29, Lys33,
	QQRLIFAG**K**QLEDGRTLSDYNI			Lys48
9	**K**IQD**K**EGIPPDQQRLIFAG**K**QLEDGRTL	39	7.43E+27	Lys29, Lys33, Lys48,
	SDYNIQ**K**ESTLHLVLRLRGG			Lys63
10	**K**EGIPPDQQRLIFAG**K**QLEDGRTLSDY	44	3.66E+23	Lys33, Lys48, Lys63
	NIQ**K**ESTLHLVLR			
11	IPPDQQRLIFAG**K**QLEDGRTLADYNIQ	20	3.38E+23	Lys48, Lys63
	**K**ESTLHLVLRLRGG			
12	NV**K**A**K**IQD**K**EGIPPDQQRLIFAG**K**QLE	36	6.15E+21	Lys27, Lys29, Lys33,
	DGRTLSDYNI			Lys48
13	**K**IQD**K**EGIPPDQQRLIFAG**K**QLEDGRT	44	5.20E+18	Lys29, Lys33, Lys48
	LSDYN			
14	**K**IQD**K**EGIPPDQQRLIFAG**K**QLEDGRT	19	2.23E+16	Lys29, Lys33, Lys48
	LAD			
15	**K**TLTG**K**TITLEVESSDTIDNV**K**A**K**IQD	19	8.01E+15	Lys6, Lys11, Lys27,
	**K**EG			Lys29, Lys33
16	MQIFV**K**TLTG**K**TITLEVEPSDTIENV**K**	25	1.17E+15	Lys6, Lys11, Lys27
17	MQIFV**K**TLTG**K**TITLEVESSDTIDNV**K**	23	8.48098E+14	Lys6, Lys11, Lys27
18	DYNIQ**K**ESTLHLVLRLRGG	62	2.40964E+11	Lys63
19	MQIFV**K**TLTG**K**TITLEVE	60	17382565255	Lys6, Lys11
20	**K**TLTG**K**TITLEVESSDTI	26	1135719784	Lys6, Lys11
21	LEVESSDTIDNV**K**	26	7757459.08	Lys27
22	TITLEVEPS	28	28304.96142	
23	**K**TLTG**K**T	67	3796.714675	Lys6, Lys11
24	DGRTLAD	23	1298.702247	
25	STLHL	69	1102.599421	
26	**K**TITL	67	315.8836468	Lys11
27	IENV**K**	38	309.1891137	Lys27
28	VEPSD	28	260.0761993	
29	TLADY	23	191.1286116	
30	IDNV**K**	29	180.0682775	Lys27

From this list of 30 statistically significant patterns obtained from the previous PD Step, the APC Step was executed with the following settings: the MERGE*Algorithm *as Global Alignment, the SIMILARITY*Score *as Hamming Distance, the TERMINATION*Condition *Score less than 0.3, the heuristics column distribution score greater than 0.3 and the minimum of three overlapping column matches. We demonstrated the efficacy of our APC Process by showing the reduced set of 9 APCs and their binding sites (Table 9).

**Table 9:** The 36 APCs of the ubiquitin Family Ranked by Standard Residual (where *m *= the number of patterns in the APC, and *n *= length of the APC)).

	APC (as regular expressions)	*m*	*n*	Quality	Coverage	Standard Residual	Binding Site
1	MQIFVKTLTGKTITLEVE[SP]S	10	76	0.31	61	4.7E+39	Lys6, Lys11,
	DTI[DE]NVKAKIQDKEGIPPDQ						Lys27, Lys29,
	QRLIFAGKQLEDGRTL[SA]DYN						Lys33, Lys48,
	IQKESTLHLVLRLRGG						Lys63
2	NVKAKIQDKEGIPPDQQRLIFAG	5	52	0.5	67	3.3E+29	Lys27, Lys29,
	KQLEDGRTL[SA]DYNIQKESTL						Lys33, Lys48,
	HLVLRLRGG						Lys63
3	MQIFVKTLTGKTITLEVEP[SP]	5	27	0.34	67	2.7E+14	Lys6, Lys11,
	DTI[ED]NVK						Lys27
4	DYNIQKESTLHLVLRLRGG	1	19	1	62	2.2E+12	Lys63
5	LEVE[SP]SDTIDNVK	2	13	0.31	54	1.0E+07	Lys27
6	KTITLEVEPS	2	10	0.4	68	4.0E+05	Lys11, Lys27
7	DGRTLADY	2	8	0.5	24	1.4E+04	
8	STLHL	1	5	1	69	1.7E+03	
9	I[ED]NVK	2	5	0.8	67	1.2E+03	Lys27

### Ubiquitin discussion

**Figure 7:**
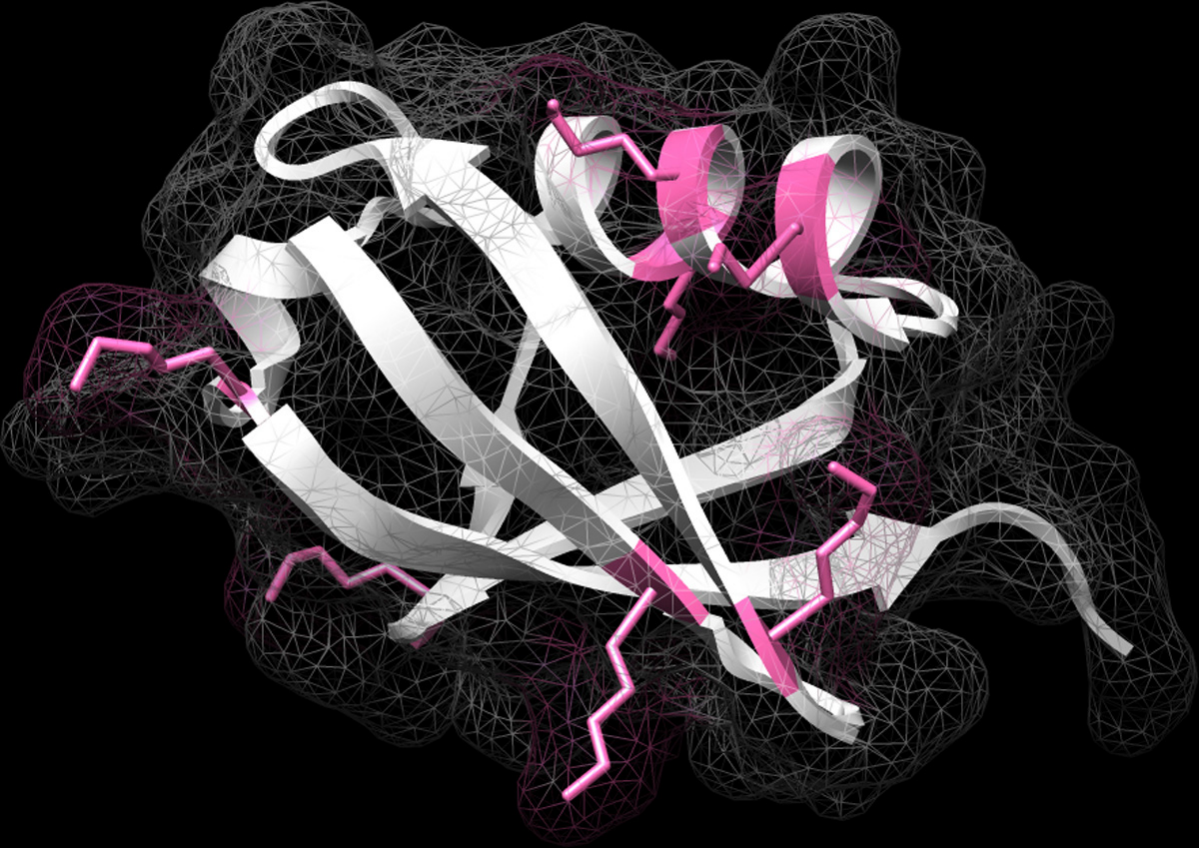
**The three-dimensional structure of the ubiquitin protein, with PDB ID 1UBQ from the protein data bank, has seven binding residues: Lys6, Lys11, Lys27, Lys29, Lys33, Lys48, and Lys63**.

The ubiquitin protein contains seven lysine residues, Lys6, Lys11, Lys27, Lys29, Lys33, Lys48, and Lys63, that can be linked to another ubiquitin to form a poly-ubiquitin chain [35-37]. The seven binding residues are visualized in the three-dimensional structure of the ubiquitin protein (Figure 7). Our resulting APCs

Figure 7: The three-dimensional structure of the ubiquitin protein, with PDB ID 1UBQ from the protein data bank, has seven binding residues: Lys6, Lys11, Lys27, Lys29, Lys33, Lys48, and Lys63.

correspond to six of the seven binding residues ()Lys6, Lys11, Lys27, Lys33, Lys48, and Lys63). The remaining Lys33 is found in an APC with only one pattern and thus stands out as a significant functional group with a distinct pattern discovered with high statistical significance in the PD Step.

For ubiquitin, our APCs are pattern alignments that agree with the emission probabilities of the pFam profile HMM (Figure 6). All eight APCs discovered agreed with the pFam HMM emission probability. Surprisingly, our results differs from PROSITE's consensus motif (PDOC00271), which missed 172 ubiquitin proteins. In drug discovery, preventing the linking of ubiquitin to its binding proteins via its binding site inhibits cancer growth.

## Conclusion

Our APC Process greatly reduces the number of APCs in comparison with other methods. This is due to the fact that the APC sstep starts with input patterns from the PD Step rather than the entire input search space. This drastically reduces the search space in a controlled manner. From the application aspect, using data from two Uniprot protein families (cytochrome c and ubiquitin), the majority of top-ranking APCs corresponded to their protein binding segments. The resulting cytochrome c binding APCs agree with the pFam emission probability. An APC represents a set of patterns as the horizontal rows and its aligned columns as the vertical columns, which can be further evaluated for amino acid conservations. In fact, for cytochrome c, the proximal and distal binding residues correspond to conserved aligned columns with R1 of 1.0. In addition, the distal APC identifies one conserved aligned column with R1 of 1.0 as the binding residue, which is not identified in PROSITE or pFam. While the ubiquitin APCs agree with pFam emission probability, six of the seven binding residues are successfully identified in the APC.

In conclusion, APCs can be used to reveal functional domains across different protein families without relying on prior knowledge or clues about the consensus regions. Currently, we are using aligned column variations as amino acid characteristics to classify protein species and gene labels. We are also extending the algorithm to discover interdependencies within APCs and long-distance associations among APCs. In more general cases of protein analysis, the function and the nature of the protein function are not clear; thus, the capability that overcomes such difficulties marks the uniqueness and novelty of our APC Process. In the broader sense, this knowledge is essential for understanding the proteins involved in epigenetics for drug discovery [38]. The development of cancer generally increases with age, and with the ageing baby-boomer population it is crucial for drug companies to find cost-effective and time-saving techniques for drug discovery.

## Competing interests

The authors declare that they have no competing interests.

## Authors' contributions

The work presented here was carried out in collaboration between all authors. AKCW defined the research theme. EAL designed methods and experiments, carried out the experiments. Both AKCW and EAL analyzed the data, interpreted the results and wrote the paper. All authors have contributed to, seen and approved the manuscript.

## Supplementary Material

Additional file 1 **The glossary of terms and mathematical notations to complement the definition in the Methodology section of this paper**. Click here for file

## Declarations

The publication costs for this article were funded by the corresponding author

This article has been published as part of *Proteome Science *Volume 11 Supplement 1, 2013: Selected articles from the IEEE International Conference on Bioinformatics and Biomedicine 2012: Proteome Science. The full contents of the supplement are available online at http://www.proteomesci.com/supplements/11/S1.
