# Supplementary material for: Ranking and compacting binding segments of protein families using aligned pattern clusters
Source: Proteome Sci. 2013 Nov 7;11(Suppl 1):S8. doi: 10.1186/1477-5956-11-S1-S8 (PMC3907781; doi:10.1186/1477-5956-11-S1-S8)
Supplement: Additional file 1 — The glossary of terms and mathematical notations to complement the definition in the Methodology section of this paper. [file 1477-5956-11-S1-S8-S1.pdf]

Glossary of Terms

An Alphabet : $\Sigma = \{\sigma_1, \sigma_2, \ldots, \sigma_{|\Sigma|-1}, \sigma_{|\Sigma|}\}$

A Sequence : $\mathbb{S} = \{s^k | k = 1, ..., |\mathbb{S}|\} = \{s^1, s^2, \ldots, s^{|\mathbb{S}|-1}, s^{|\mathbb{S}|}\}$

A Set of Unaligned Pattern : $\bar{\mathbb{P}} = \{\bar{p}^i | i = 1, ..., |\bar{\mathbb{P}}|\} = \{\bar{p}^1, \bar{p}^2, \ldots, \bar{p}^{|\bar{\mathbb{P}}|-1}, \bar{p}^{|\bar{\mathbb{P}}|}\}$

An Unaligned Pattern : $\bar{p}^i = s^i_1 s^i_2 ... s^i_{|\bar{p}^i|}$

A Set of Aligned Pattern : $\mathbb{P} = \{p^i | i = 1, ..., |\mathbb{P}|\} = \{p^1, p^2, \ldots, p^{|\mathbb{P}|-1}, p^{|\mathbb{P}|}\}$

The Occurrence of the Pattern $\bar{p}^i$: $occ(\bar{p}^i) = j_i$ such that $\bar{p}^i = s^i_{j_i} s^i_{j_i+1} \ldots s^i_{j_i+|\bar{p}^i|-1}$, where i is the index of the sequence that pattern occurs in, and j_i is the starting index the pattern in that sequence.

A Set of APC : $\mathbb{C} = \{C^l | l = 1, ..., |\mathbb{C}|\} = \{C^1, C^2, \ldots, C^{|\mathbb{C}|-1}, C^{|\mathbb{C}|}\}$

An APC :

$$
\begin{aligned}
C^l &= \texttt{ALIGN}(\mathbb{P}^l), && (1)\\
&= \begin{pmatrix} s^1_1 & s^1_2 & \ldots & s^1_n \\ s^2_1 & s^2_2 & \ldots & s^2_n \\ \vdots & \vdots & \vdots & \vdots \\ s^m_1 & s^m_2 & \ldots & s^m_n \end{pmatrix}_{m\times n} = \begin{pmatrix} p^1 \\ p^2 \\ \vdots \\ p^m \end{pmatrix}, && (2)\\
&= \begin{pmatrix} c_1 & c_2 & \ldots & c_n \end{pmatrix}. && (3)
\end{aligned}
$$

where $s^i_j \in \Sigma \cup \{-\} \cup \{*\}$ is an pattern p^i with a newly aligned column index j. Each of the $|\mathbb{P}^l| = m$ patterns in the rows of C^l is of length $|C^l| = n$.

An Aligned Pattern : $p_i = s^i_1 s^i_2 ... s^i_{|p_i|}$ is a subsequence of order-preserving elements maximizing the similarity of the patterns against a set of pattern from APC, $\mathbb{P}_l$, with gaps, wildcards, and mismatches to the length $|\mathbb{P}^l| = n$.

An Aligned Column: Let c_j in C^l represents the jth column of amino acids from the set of patterns that forms the current APC, $C^l = \big(c_1, c_2, \ldots, \backslash\big)$.

Data Induced by the Unaligned Pattern Let $\mathbb{D}(\bar{p}^i)$, be all the occurrences of the pattern, $\bar{p}^i$, that is in the input sequence. We call $\mathbb{D}(\bar{p}^i)$ the data induced by $\bar{p}^i$ or the induced data of $\bar{p}^i$. We will return to the concept for APC which is later used for computing the measures for aligned columns.

Data Induced by APC Let $\mathbb{D}(C^l)$ be data induced by the APC C^l, which is the subset of segments from the input sequences, or the data subspace containing all the pattern from the APC, C^l, $\mathbb{P}^l = \{p^1, p^2, \ldots, p^m\}^T$. We call $\mathbb{D}(C^l)$ the data induced by C^l or the induced data of C^l. Then $\mathbb{D}(C^l)$ is then the union of the segements from the input sequences induced by all the patterns contained in C^l, $\mathbb{D}(C^l) = \mathbb{D}(p^1) \cup \mathbb{D}(p^2) \cup \cdots \cup \mathbb{D}(p^m) = \bigcup_{\forall p^i \in \mathbb{P}^l} \mathbb{D}(p^i)$
